# Trade‐off between fecundity and survival generates stabilizing selection on gall size

**DOI:** 10.1002/ece3.6682

**Published:** 2020-08-17

**Authors:** Amanda K. Weaver, Glen Ray Hood, Michael Foster, Scott P. Egan

**Affiliations:** ^1^ Department of BioSciences Rice University Houston TX USA; ^2^ Department of Biological Sciences Wayne State University Detroit MI USA

**Keywords:** Cynipidae, gall former, Hymenoptera, multitrophic interactions, natural selection

## Abstract

Complex interactions within multitrophic communities are fundamental to the evolution of individual species that reside within them. One common outcome of species interactions are fitness trade‐offs, where traits adaptive in some circumstances are maladaptive in others. Here, we identify a fitness trade‐off between fecundity and survival in the cynipid wasp *Callirhytis quercusbatatoides* that induces multichambered galls on the stem of its host plant *Quercus virginiana*. We first quantified this trade‐off in natural populations by documenting two relationships: a positive association between the trait gall size and fecundity, as larger galls contain more offspring, and a negative association between gall size and survival, as larger galls are attacked by birds at a higher rate. Next, we performed a field‐based experimental evolution study where birds were excluded from the entire canopy of 11 large host trees for five years. As a result of the five‐year release from avian predators, we observed a significant shift to larger galls per tree. Overall, our study demonstrates how two opposing forces of selection can generate stabilizing selection on a critical phenotypic trait in wild populations, and how traits can evolve rapidly in the predicted direction when conditions change.

## Graphical abstract

Complex interactions within multitrophic communities can result in fitness trade‐offs for individual species, where traits adaptive in some circumstances are maladaptive in others. In this paper, we identify a fitness trade‐off in the cynipid wasp *Callirhytis quercusbatatoides*, which induces multichambered galls on the stem of its host plant *Quercus virginiana*. We find a positive association between the trait “gall size” and fecundity, where larger galls contain more offspring, and a negative association between gall size and survival, where larger galls are attacked by birds at a higher rate. We then use a field‐based experimental evolution study where birds were excluded from host trees for five years and observe a significant shift to larger galls per tree in the direction predicted by natural selection.

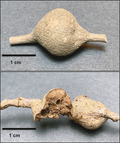

## 1 INTRODUCTION

The complex interactions within multitrophic communities are fundamental components of ecosystems (Seibold, Cadotte, MacIvor, Thorn, & Müller, 2018), which can have direct effects on populations (Hood, Comerford, Weaver, Morton, & Egan, 2019), and indirect effects on species interactions that influence entire communities (Raupp, Shrewsbury, & Herms, 2010; Rogers, Hille Ris Lambers, Miller, & Tewksbury, 2012). Species interactions can contribute to natural selection on genetically based traits within populations (Endler, 1986) that can have indirect effects on other members of the community (Hendry, 2017). If selection changes across space and/or time due to changes in species interactions, this can have cascading effects driving eco‐evolutionary dynamics in local communities. Specific to the current study, changes in selection can lead to trade‐offs, where the costs and benefits of phenotypes change under different environmental conditions (Hendry, 2017; Start, Weis, & Gilbert, 2019).

Trade‐offs can be common and widespread in nature (Agrawal, Conner, & Rasmann, 2010; Roff & Fairbairn, 2007), such as those observed between egg size versus egg number in insects (e.g., Berrigan, 1991), while others are species‐specific, such as the negative association between reproduction and mate calling in monkeys (Dunn et al., 2015). Interspecific interactions can also lead to trade‐offs, such as when ant tending deters harmful herbivores, but also beneficial pollinators (Ohm & Miller, 2014). In addition, changes in when, where, or how species interact can also lead to trade‐offs. For example, when the shape and magnitude of phenotypic selection of an extended insect phenotype is altered across an urban–rural gradient due to differences in predation pressure (Hood et al., 2019; Start, Bonner, Weis, & Gilbert, 2018).

Over the last few centuries, >80% of the Earth's land surface has been modified to grow crops, raise animals, obtain resources, and build structures (Sanderson et al., 2002). This rapid and broad environmental change disrupts species interactions, which can alter the abundance of individuals within populations (Hood et al., 2019) and result in patterns of species loss (Fischer & Lindenmayer, 2007; Raupp et al., 2010; Rogers et al., 2012). Moreover, habitat modification is often associated with change in predator density, particularly in insectivorous birds (e.g., woodpeckers), which generally exhibit smaller group sizes in highly fragmented landscapes (Conner & Rudolph, 1991). While changes in species interactions have important ramifications for ecology, they can also promote evolutionary change, often quite rapidly. Recent examples of contemporary evolution in response to environmental change include industrial melanism (Cook & Saccheri, 2013), resistance to heavy metals, pesticides, and toxins (Reznick & Ghalambor, 2001), and phenological shifts due to climate change (Parmesan, 2006). More generally, a recent review by Johnson and Munshi‐South (2017) highlighted other examples of heritable evolutionary change in response to anthropogenic change.

The biology and natural history of gall‐forming wasps in the family Cynipidae (Hymenoptera) facilitate studies of natural selection (Egan, Hood, & Ott, 2011; Price, Abrahamson, Hunter, & Melika, 2004). These wasps induce tumor‐like outgrowths of plant material that are controlled, in part, by the gall wasp (Stone, Schönrogge, Atkinson, Bellido, & Pujade‐Villar, 2002). Gall wasps are sessile during development inside the gall, easily located, and the fates of individuals within galls are readily monitored and associated with gall characteristics, such as size, shape, and color (Craig, Itami, & Horner, 2007; Egan et al., 2011; Heath, Abbot, & Stireman, 2018; Hood & Ott, 2010; Start et al., 2018). Galls, which house developing larvae, represent extended phenotypes of the gall former (Dawkins, 1982). Thus, the gall itself reflects the interaction of insect and plant genomes and the environment and thus gall phenotypes (e.g., gall size) are thought to be both heritable and plastic (László & Tóthmérész, 2013; Weis & Abrahamson, 1985, 1986; Weis & Gorman, 1990). Gall size in many systems has a well‐documented ecological role, contributing to defense against natural enemies (Hood, Zhang, & Egan, 2018; Start et al., 2018; Stone & Schönrogge, 2003), and being positively correlated with adult size, and potential fecundity (Ito & Hijii, 2004). In many species of gall formers, including the species studied here, gall growth is maintained by active larval feeding (Stone et al., 2002); thus, at the outset of the growing season, final gall size is a record of the size attained at the completion of feeding prior to pupation or the size attained by the time larvae succumb to the effects of natural enemies, host plant defenses, and/or pathogens. Consequently, gall size can be tested for association with the probability of survival (adult emergence) in the presence (Craig et al., 2007) or absence of natural enemies using experimental exclusions (Egan et al., 2011).

Herein, we estimate patterns of phenotypic selection on the ecologically important trait “gall size” for the cynipid wasp *Callirhytis quercusbatatoides* on the stems of its host plant, the southern live oak, *Quercus virginiana*. In this system, gall size is associated with a potential trade‐off in fitness: larger galls generate more offspring, but are attacked by birds more often. We first present observational studies that estimate the association between gall size and chamber number, a proxy for fecundity, in the absence of bird predation. We then measure the association between gall size and the probability of predation by birds, an important, albeit understudied, source for gall former mortality (Hails & Crawley, 1992; László, Sólyom, Prázsmári, Barta, & Tóthmérész, 2014; Schönrogge, Begg, & Stone, 2013; Tscharntke, 1992). Next, following Mitchell‐Olds and Shaw (1987), which recommended experimental manipulation to accompany observational analysis of selection, we performed a manipulative experiment that excluded predation by birds from the canopy of 11 large live oak trees to test for the effects of relaxed directional selection on gall size based on our predictions from natural populations. Overall, when birds were excluded for five years, we found evidence for a predictable shift in gall size in response to relaxed selection.

## 2 METHODS

### 2.1 Study system

**Figure 1:**
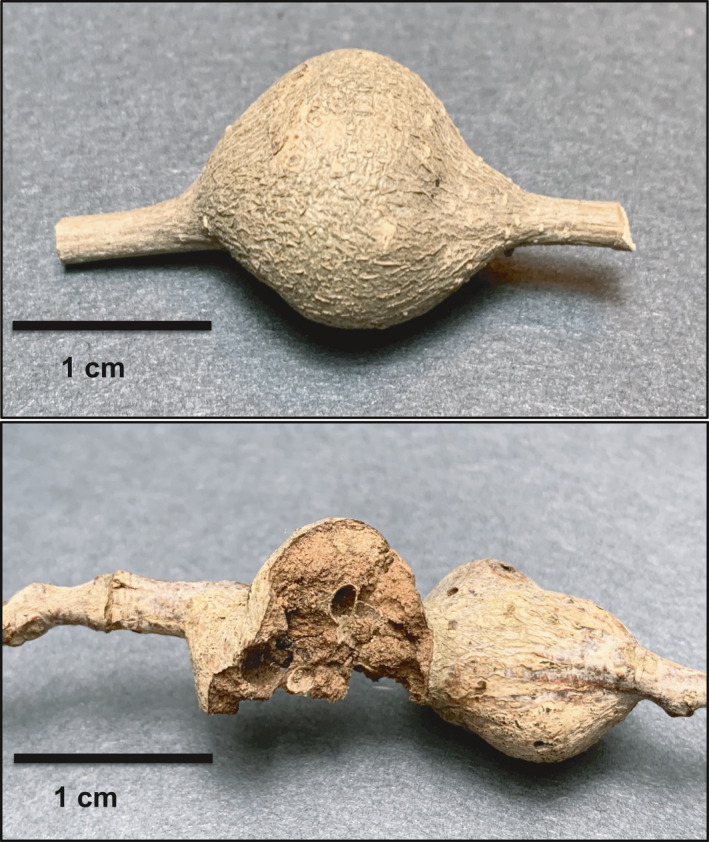
Multichambered stem galls induced by the cynipid wasp *Callirhytis quercusbatatoides* on its host plant, the southern live oak, *Quercus virginiana*. Top panel: intact gall; bottom panel: characteristic attack by birds

Gall wasps (Hymenoptera: Cynipidae: Cynipini) often exhibit a cyclically parthenogenic life cycle, alternating between an asexual and sexual generation (Stone et al., 2002). The asexual generation of the wasp *Callirhytis quercusbatatoides*, which is the focus of the current study, develops within multichambered ellipsoid‐shaped galls (Figure 1) induced on newly growing stems of the southern live oak, *Quercus virginiana* (Ashmead, 1881). The sexual generation of this species is unknown, which includes this generation's oviposition behavior that induces the asexual generation galls studied here. Most cynipids, including *C. quercusbatatoides*, are small (2–3 mm), harbor hundreds to thousands of eggs (Hood & Ott, 2017), and are poor fliers. As a result, females likely oviposit multiple times on different locations within a tree, and disperse less often between trees (Egan & Ott, 2007).

The stem galls harboring the asexual generation develop in late summer through winter and generate a highly variable number of adult females based on the size of the gall and the number of chambers inside (i.e., larger galls have more chambers inside and generate more wasps). During the winter months when galls reach their penultimate size and fewer invertebrates are available, asexual generation galls are commonly attacked by insectivorous birds, most commonly by the downy woodpecker (*Dryobates pubescens*), which is native and common in the study region. On multiple occasions, all four authors on this study have independently observed *D. pubescens* predating *C. quercusbatatoides* galls at our field sites.

The galls induced by *C. quercusbatatoides* can remain on trees for several years, but old and new galls are easy to differentiate based on color and insect use. Newly induced galls are a vibrant silver or gray and do not contain emergence holes or evidence of predation, while older galls are darker, with mold and lichens, and typically contain emergence holes and predation markings. We focused our study on galls induced in the current year for our study Important to the present study, birds leave characteristic damage where they chisel out insects with their beaks (Figure 1). Galls induced by several other species on *Q. virginiana* in our study area are attacked by both birds and other vertebrate predators, such as squirrels, mice, and rats, which leave distinct teeth markings. The *C. quercusbatatoides* stem galls only showed evidence of beak damage consistent with birds and were further verified by inspecting galls directly observed to be attacked by birds to inform our search.

The asexual generation galls of *C. quercusbatatoides* also harbor a community of invertebrate natural enemies, including five species of parasitoid wasps in the following three families: Ormyridae, Eurytomidae, and Eupelmidae (Noyes, 2019; C. Davis, L. Zhang, S.P. Egan, unpublished data). Additionally, a wood boring moth, the oakgall clearwing, *Synanthedon decipiens* (Lepidoptera: Sesiidae), feeds on internal gall tissue as a larva that likely result in *C. quercusbatatoides* death (Engelhardt, 1946). The effect of the invertebrate natural enemy community was not the focus of this current study.

### 2.2 Sampling natural populations and gall measurements

When *C. quercusbatatoides* galls matured in early spring of 2016, we haphazardly harvested 1,440 mature galls from 33 individual host trees located throughout the Lynn R. Lowrey Arboretum encompassing a 1.2 km^2^ area located throughout the campus of Rice University in Houston, Texas, USA (http://arboretum.rice.edu). Galls were collected from the mid and lower portion of the canopy of each live oak where they are most common, using a 2‐m ladder, or a 5‐m pole pruners. The number of gall harvest by tree loosely tracks gall density per tree, which is highly variable (see Table 1).

**Table 1:** Mean gall size (±*SE*) estimated by measuring length (mm), width (mm), and volume (cm^3^), and the percent of galls predated by birds (% P) for *C. quercusbatatoides* on 33 individual live oak trees in the Lynn R. Lowrey Arboretum located throughout the campus of Rice University in Houston, Texas, USA (*N* = number of galls sampled per tree; CV = coefficient of variation)

Tree	*N*	Length ± *SE* (range)	Length CV	Width ± SE (range)	Width CV	Volume ± *SE* (range)	Volume CV	% P
T1	29	18.34 ± 0.93 (9.5–26.8)	0.27	15.83 ± 0.69 (7.2–21.7)	0.23	2.71 ± 0.30 (0.29–6.03)	0.59	24
T2	34	15.86 ± 0.81 (10.4–34.6)	0.30	12.26 ± 0.37 (7.57–17.12)	0.18	1.36 ± 0.14 (0.40–3.67)	0.59	21
T3	33	17.84 ± 0.96 (12.3–38.6)	0.31	14.00 ± 0.45 (10.15–21.75)	0.18	1.97 ± 022 (0.72–6.01)	0.61	36
T4	29	21.25 ± 1.54 (12.90–48.95)	0.38	16.27 ± 0.66 (10.95–22.79)	0.21	3.27 ± 0.44 (0.95–11.78)	0.71	24
T5	79	14.17 ± 0.32 (8.72–24.20)	0.20	15.26 ± 0.45 (7.57–25.42)	0.26	1.97 ± 0.15 (0.34–7.21)	0.67	16
T6	23	18.73 ± 1.27 (8.85–36.60)	0.33	16.24 ± 0.97 (8.60–26.77)	0.28	2.93 ± 0.45 (0.34–8.62)	0.74	35
T7	33	17.21 ± 1.44 (9.77–39.41)	0.47	14.03 ± 0.70 (8.65–27.46)	0.27	2.36 ± 0.54 (0.38–15.56)	1.25	48
T8	35	15.47 ± 0.72 (8.60–23.49)	0.25	14.81 ± 0.74 (9.20–22.22)	0.25	2.02 ± 0.24 (0.45–3.67)	0.56	60
T9	39	18.17 ± 1.29 (7.90–45.66)	0.44	14.34 ± 0.63 (6.40–23.46)	0.27	2.36 ± 0.37 (0.17–11.63)	0.96	15
T10	39	17.64 ± 1.03 (7.69–34.85)	0.35	16.48 ± 0.97 (9.43–26.59)	0.27	3.02 ± 0.61 (0.36–12.76)	0.93	64
T11	61	15.78 ± 0.61 (9.06–31.32)	0.30	13.51 ± 0.45 (5.66–21.68)	0.24	1.62 ± 0.14 (0.18–5.04)	0.63	28
T12	30	20.44 ± 1.23 (11.91–41.21)	0.32	14.95 ± 0.74 (8.76–19.00)	0.19	2.35 ± 0.35 (0.48–5.57)	0.57	67
T13	47	16.88 ± 0.61 (8.79–26.82)	0.25	13.93 ± 0.60 (8.99–29.19)	0.26	1.84 ± 0.26 (0.48– 9.77)	0.85	60
T14	28	18.50 ± 1.00 (10.00–31.08)	0.29	15.15 ± 0.96 (8.02–28.41)	0.34	2.72 ± 0.49 (0.38–12.23)	0.95	14
T15	58	17.32 ± 0.62 (8.69–30.77)	0.27	15.54 ± 0.48 (7.21–27.06)	0.22	2.40 ± 0.21 (0.24– 8.47)	0.62	19
T16	21	19.86 ± 1.60 (6.66–35.55)	0.37	14.28 ± 0.96 (5.45–24.76)	0.31	2.68 ± 0.58 (0.10–11.41)	0.99	0
T17	41	19.45 ± 0.98 (11.22–35.31)	0.32	16.33 ± 0.59 (8.82–26.08)	0.21	2.95 ± 0.27 (0.50–7.01)	0.55	31
T18	80	12.54 ± 0.31 (6.84–18.80)	0.22	11.33 ± 0.48 (5.43–22.00)	0.34	1.03 ± 0.10 (0.13–3.34)	0.81	30
T19	35	19.38 ± 1.60 (9.06–46.56)	0.49	14.70 ± 0.87 (7.62–25.89)	0.33	2.66 ± 0.50 (0.31–11.16)	1.06	20
T20	20	15.73 ± 1.40 (7.39–30.73)	0.40	13.92 ± 0.85 (6.64–19.20)	0.26	1.78 ± 0.31 (0.17–4.54)	0.73	20
T21	66	14.74 ± 0.60 (6.66–35.05)	0.33	13.30 ± 0.47 (6.93–20.15)	0.26	1.60 ± 0.60 (0.18–6.43)	0.75	29
T22	57	19.23 ± 0.80 (10.78–34.58)	0.31	14.87 ± 0.60 (8.95–28.12)	0.28	2.64 ± 0.33 (0.48–10.08)	0.88	44
T23	34	19.10 ± 1.20 (7.60–39.89)	0.36	15.44 ± 0.78 (8.00–25.78)	0.27	2.69 ± 0.37 (0.30–9.81)	0.74	35
T24	85	15.71 ± 0.73 (7.65–49.29)	0.41	14.33 ± 0.60 (6.89–28.34)	0.29	1.70 ± 0.74 (0.23–9.82)	0.92	47
T25	21	19.77 ± 1.48 (11.25–35.25)	0.33	15.70 ± 1.09 (9.39–25.14)	0.26	3.00 ± 0.74 (0.58–11.48)	0.92	43
T26	31	13.50 ± 0.55 (8.29–21.05)	0.23	13.38 ± 0.65 (7.66–22.12)	0.26	1.45 ± 0.21 (0.25–5.35)	0.76	13
T27	46	15.91 ± 0.72 (6.89–31.97)	0.30	10.39 ± 0.80 (5.08–18.92)	0.33	1.15 ± 0.30 (0.09–5.99)	1.13	61
T28	57	16.46 ± 0.65 (9.18–31.40)	0.30	16.55 ± 0.65 (8.20–26.63)	0.28	2.74 ± 0.28 (0.36–7.46)	0.73	18
T29	49	18.06 ± 0.63 (10.42–33.79)	0.24	13.52 ± 0.52 (7.18–18.72)	0.22	1.78 ± 0.15 (0.35–3.81)	0.49	47
T30	43	17.45 ± 0.81 (7.78–28.35)	0.30	13.25 ± 0.62 (5.33–21.14)	0.29	1.95 ± 0.24 (0.14–6.00)	0.77	14
T31	69	17.40 ± 0.66 (7.76–31.08)	0.31	13.20 ± 0.41 (5.81–20.87)	0.24	1.79 ± 0.15 (0.16–5.32)	0.68	10
T32	30	17.40 ± 0.92 (8.73–28.00)	0.28	14.95 ± 0.84 (5.20–24.22)	0.29	2.36 ± 0.37 (0.12–7.52)	0.79	30
T33	57	18.45 ± 0.95 (9.25–36.26)	0.39	15.36 ± 0.66 (8.23–25.97)	0.28	2.62 ± 0.33 (0.35–10.90)	0.83	35
All trees	1,440	16.96 ± 0.16 (6.66–49.29)	0.35	14.37 ± 0.12 (5.08–29.19)	0.28	2.14 ± 0.05 (0.09–15.56)	0.85	32

We initially considered three different measures of gall size: length, width, and volume. However, the width of galls with heavy bird predation was often more difficult to measure than length, making volume equally difficult to calculate for all bird‐attacked galls. In addition, both width and volume were highly correlated with gall length (length × width: *r* = 0.6472, *p* < .0001; length × volume: *r* = 0.7698, *p* < .0001) and, similarly, predicted gall chamber number (width: *r* = 0.6472, *p* < .0001; volume: *r* = 0.7698, *p* < .0001) and thus generating qualitatively similar results. As such, we present fitness estimates as a function of gall length, but list gall size values and summary statistics for width and volume in Tables 1 and 2 for comparison.

**Table 2:** Mean gall size (±*SE*) estimated by measuring length (mm), width (mm), and volume (cm^3^), and the percent of galls predated by birds (% P) for *C. quercusbatatoides* on 11 individual live oak trees where birds had been excluded in the Texas Medical Center located in Houston, Texas, USA (*N* = number of galls sampled per tree; CV = coefficient of variation)

Netted Tree	*N*	Length ± *SE* (range)	Length CV	Width ± *SE* (range)	Width CV	Volume ± *SE* (range)	Volume CV	% P
NT1	53	18.74 ± 0.94 (9.24–35.54)	0.37	17.86 ± 0.85 (8.56–32.22)	0.35	4.14 ± 0.55 (0.36–19.32)	0.97	15
NT2	79	18.03 ± 0.75 (9.24–35.54)	0.37	15.74 ± 0.57 (6.30–26.16)	0.32	2.99 ± 0.29 (0.20–11.25)	0.86	5
NT3	174	17.48 ± 0.43 (5.90–35.56)	0.32	14.83 ± 0.31 (4.77–31.02)	0.28	2.39 ± 0.16 (0.07–12.68)	0.86	5
NT4	115	19.71 ± 0.79 (7.16–44.70)	0.43	14.53 ± 0.40 (6.54–28.74)	0.30	2.76 ± 0.26 (0.17–18.00)	1.01	0.8
NT5	66	24.28 ± 1.31 (11.11–54.82)	0.44	21.86 ± 1.04 (8.29–43.53)	0.39	9.04 ± 1.33 (0.41–54.39)	1.19	12
NT6	32	16.69 ± 0.90 (8.51–32.35)	0.31	15.74 ± 0.68 (6.80–25.39)	0.24	2.49 ± 0.34 (0.23–10.92)	0.78	6
NT7	63	25.26 ± 1.14 (9.82–43.50)	0.36	17.88 ± 0.60 (9.33–31.21)	0.27	5.08 ± 0.53 (0.52–22.09)	0.83	5
NT8	75	16.81 ± 0.51 (9.38–35.39)	0.26	16.41 ± 0.51 (8,02–27.35)	0.27	2.75 ± 0.24 (0.39–9.85)	0.74	12
NT9	93	18.19 ± 0.73 (8.24–38.15)	0.39	16.41 ± 0.54 (6.60–29.63)	0.32	3.29 ± 0.36 (0.24–17.48)	1.06	2
NT10	61	22.53 ± 1.01 (12.48–51.83)	0.35	17.31 ± 0.64 (10.21–31.04)	0.29	4.43 ± 0.59 (0.74–25.53)	1.04	2
NT11	129	17.45 ± 0.50 (7.63–36.72)	0.33	16.78 ± 0.41 (5.62–29.13)	0.28	3.01 ± 0.21 (0.17–15.00)	0.80	2
All Netted Trees	940	19.14 ± 0.24 (5.90–54.82)	0.39	16.44 ± 0.17 (4.77–43.53)	0.32	3.53 ± 0.14 (0.07–54.39)	1.20	5

Gall size was estimated by making a linear measurement from the tips of the ellipsoid‐shaped swelling running parallel to the branch to which it was attached (Figure 1) using digital calipers to the nearest 0.01 mm. The multichambered stem galls induced by female *C. quercusbatatoides* vary in size and the number of chambers per gall and, thus, the number of offspring per gall. To test the association between gall size and wasp fitness in the absence of predation, we sampled and carefully dissected 101 galls that had not been attacked by birds that spanned the range of sizes observed in this study (size range: 8.33–33.21 mm in length) distributed across the 33 trees. This is an exceptionally time limiting step, as each gall is constructed of hard stem wood. Thus, we soaked galls in water for 24 hr before dissection to soften the tissue before making ~2‐mm cross sections with a razor blade to accurately quantify chamber number per gall. By measuring the size and chamber number of galls that had not been attacked by birds, we were able to estimate the association of fitness and gall size based on the interaction between the wasp and host plant in the absence of predation.

To test the association between survivorship and gall size due to bird predation, we compared the relationship between gall size to the probability of *C. quercusbatatoides* survival (= inverse of probability of predation from bird attack) across all 33 trees and 1,440 galls sampled. Each gall was inspected for physical damage characterized by chiseled markings consistent with beak damage caused by the birds foraging for larval insects inside the gall (see Figure 1). Since galls attacked by birds cause complete or near complete mortality of individuals within a multichambered stem gall, survivorship from bird predation is measured as a binomial variable (1 = survive, 0 = death).

### 2.3 Testing the association between gall size and measures of fitness

To visualize the association between gall size and fitness, we fit a nonparametric cubic spline (Schluter, 1988) to the relationship between gall size and (a) fecundity (chamber number) per gall and (b) survivorship (bird predation) using the program *mgcv* in R (version 3.5.2). In the analysis of predation, our sample size was large enough that we fit a cubic spline that included individual tree as a covariate. Uncertainty in the fit, generated by sample size variation, is captured in the standard errors generated by the Bayesian method.

Using these associations between phenotype and fitness, we characterized the linear and potential nonlinear components of fitness using regression analysis (Lande & Arnold, 1983). The number of chambers per gall was measured as a continuous variable, where standard linear regression could be used for significance testing. However, bird predation was a binomial variable; thus, we used a logistic regression followed by log‐likelihood tests to determine the significance of regression terms for this variable (Brodie, Moore, & Janzen, 1995; Janzen & Stern, 1998). In both analyses, gall size was standardized to a mean of 0 and a standard deviation of 1 so that regression coefficients, when appropriately transformed, would equate to standard selection gradients. Although logistic regression provides appropriate tests of significance for categorical data (Brodie & Janzen, 1996), the resultant linear and nonlinear coefficients cannot be directly compared with standard derived selection gradients (Janzen & Stern, 1998) or used to predict evolutionary responses to selection (Lande, 1979). Thus, we used an SAS script (http://www.public.iastate.edu/∼fjanzen/homepage.html) to transform the logistically derived regression coefficient into an approximate selection gradient readily comparable with traditional approaches to measuring selection (Egan et al., 2011; Janzen & Stern, 1998).

### 2.4 Manipulative experiment excluding bird predation

Based on the results of our observational study, we tested the hypothesis that an experimental release from selection by birds would result in an evolutionary increase in average *C. quercusbatatoides* gall size. Adjacent to the Lynn R. Lowrey Arboretum at Rice University, in an area of the Texas Medical Center and associated office buildings, the entire canopies of mature live oaks (*Q. virginiana*) have been individually and completely covered in netting for five years to deter the nesting of birds (see Hood et al., 2019a for further details). This scenario provides a “natural experiment” to test the impacts of bird predation on gall size. The location of these protected trees was <1 km from the edge of Rice University where our observational study took place, and trees at both sites growing in similar semiurban conditions and approximately equidistant to buildings, sidewalks, roads, and other man‐made structures, suggesting “site” has a minimal influence on our results. We found 11 netted trees that had significant densities of *C. quercusbatatoides* (most gall‐forming species are naturally patchy within populations of its host plant; e.g., Egan & Ott, 2007). We harvested 940 galls across these 11 trees using the same methods described above. The presence of bird netting significantly lowered bird predation (netted trees: mean bird predation on galls per tree ± *SE* =6 ± 3%; non‐netted trees: mean bird predation ± *SE* =32 ± 3%; *X* ^2^_df=42_ = 247.2, *p* < .00001; Table 1, 2). The galls attacked in the netted trees were always found on the edges of the netting within the reach of birds. The length of these 940 galls was measured using the same methods described above. We then compared gall size of *C. quercusbatatoides* from these 11 netted trees to the 33 non‐netted trees located at the nearby Lynn R. Lowrey Arboretum, which are under natural levels of bird predation, using a nested ANOVA with predator treatment (birds present versus absent) and individual tree nested within predator treatment as fixed factors.

In addition, we quantified the observed evolutionary change in gall phenotypes over the five‐year field experiment as darwins (Haldane, 1949), a unit of evolutionary change in a trait expressed in terms of factors of the constant *e* (=2.71828) over one million years, and as haldanes (Gingerich, 1993), a unit of evolutionary change expressed in terms of the phenotypic standard deviations per generation (Hendry & Kinnison, 1999). To calculate darwins, we used the equation: *r* = (ln*X*_2_ − ln*X*_1_)/Δ*t*, where *X*_1_ and *X*_2_ are the initial and final values of the trait and Δ*t* is the change in time in millions of years. To calculate haldanes, we used the equation: *H* = [(*X*_1_/*s*_p_) − (*X*_1_/*s*_p_)]/ *g*, where *X*_1_ and *X*_2_ are the initial and final values of the trait, *s*_p_ is the pooled standard deviation [=((*n*_1_ − 1)(*s*_1_)^2^ + (*n*_2_ − 1)(*s*_2_)^2^)/(*n*_1_ + *n*_2_ − 2)], and *g* is the time interval between samples counted in the number of generations. We calculated darwins and haldanes as a point estimate comparing the average observed across the 11 trees excluding bird predation versus trees under natural conditions (see Figure 4). These measurements assume that the gall wasps from bird exclusion experiment and natural conditions had a common origin in the past (see Hendry & Kinnison, 1999) and that the changes in phenotype we observed were at least partially genetically controlled.

## 3 RESULTS

### 3.1 Gall measurements under natural conditions

For the 1,440 galls collected across the 33 trees, mean gall length ± *SE* was 16.96 ± 0.16 mm and ranged in length from 6.66 to 49.29 mm (Table 1). Bird predation was also highly variable, with a mean predation rate per tree ± *SE* of 32% ± 3% and ranged from 0% to 67% per tree (Table 1). For the subsample of 101 nonpredated galls that were dissected to count the number of chambers, the mean chamber number per gall was 14.58 ± 1.56 *SE*, ranging between 1 and 77 chambers. We used these measurements to test for associations between gall size and chamber number (fecundity) or gall size and bird predation (survival).

### 3.2 Association between gall size and chamber number

**Figure 2:**
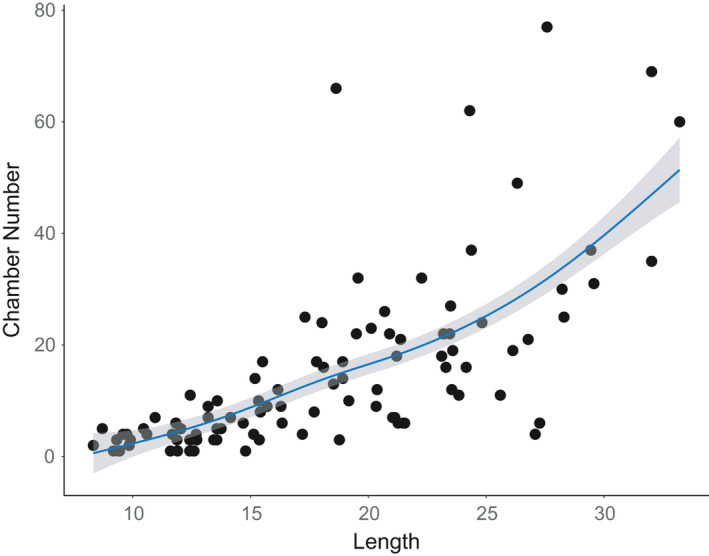
Association between gall size (length) and chamber number from 101 dissected *C. quercusbatatoides* galls fit with a cubic spline (solid line) ± *SE* (shaded region)

The relationship between gall size (length) and chamber number, a proxy for fitness in the absence of bird predation, was highly significant (*R* ^2^ = 0.47, *F*_df=1, 99_ = 89.49, *p* < .0001; Figure 2). The addition of a nonlinear term did not explain any additional variation (*p* > .50). However, the slope of the linear relationship, which estimates the selection gradient on gall size was 0.74 ± 0.08 *SE*. This estimate of directional selection serves to quantify the association between fitness and gall size in the absence of predation. Thus, the observed significant positive selection gradient suggests strong directional selection for larger galls and thus greater chamber number in the absence of bird predation.

### 3.3 Association between gall size and survival

**Figure 3:**
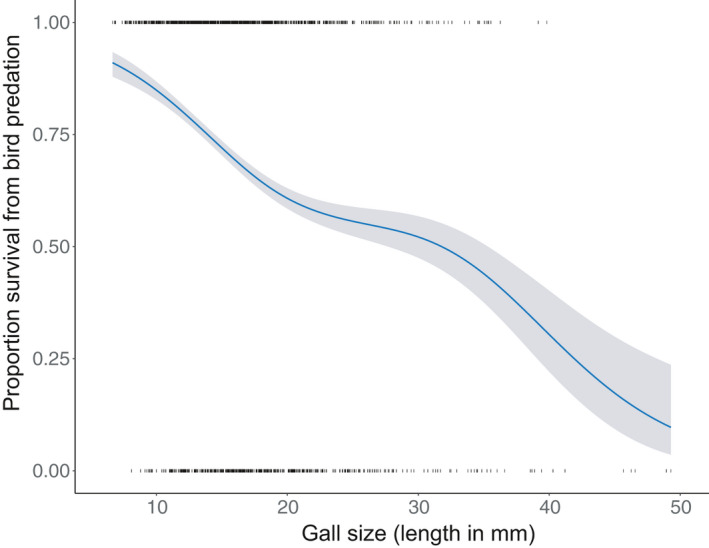
Association between gall size (length) and survival from bird attack (0 = no; 1 = yes) for 1,440 *C. quercusbatatoides* galls across 33 *Q. virginiana* trees fit with a cubic spline (solid line) ± *SE* (shaded region). Survival from bird predation for each gall is illustrated by tick marks

When all 1,440 galls from all 33 trees were pooled to estimate selection across the population, the logistic regression describing the relationship between gall size and the probability of *C. quercusbatatoides* survivorship revealed that the linear term was significant (χ^2^_df=1_ = 6.09, *p* = .0136; Figure 3) and the nonlinear term did not explain any additional variation (*p* = .40). The slope of this linear relationship, which estimates the selection gradient on gall size based on survival was −0.09 ± 0.01 *SE*. This estimate of negative directional selection serves to quantify the association between fitness and gall size in the presence of bird predation. Thus, the observed significant negative selection gradient suggests weak directional selection favoring increased survival of wasps in smaller galls due to predation of larger galls by birds (Figure 3).

### 3.4 Phenotypic response to bird exclusion

**Figure 4:**
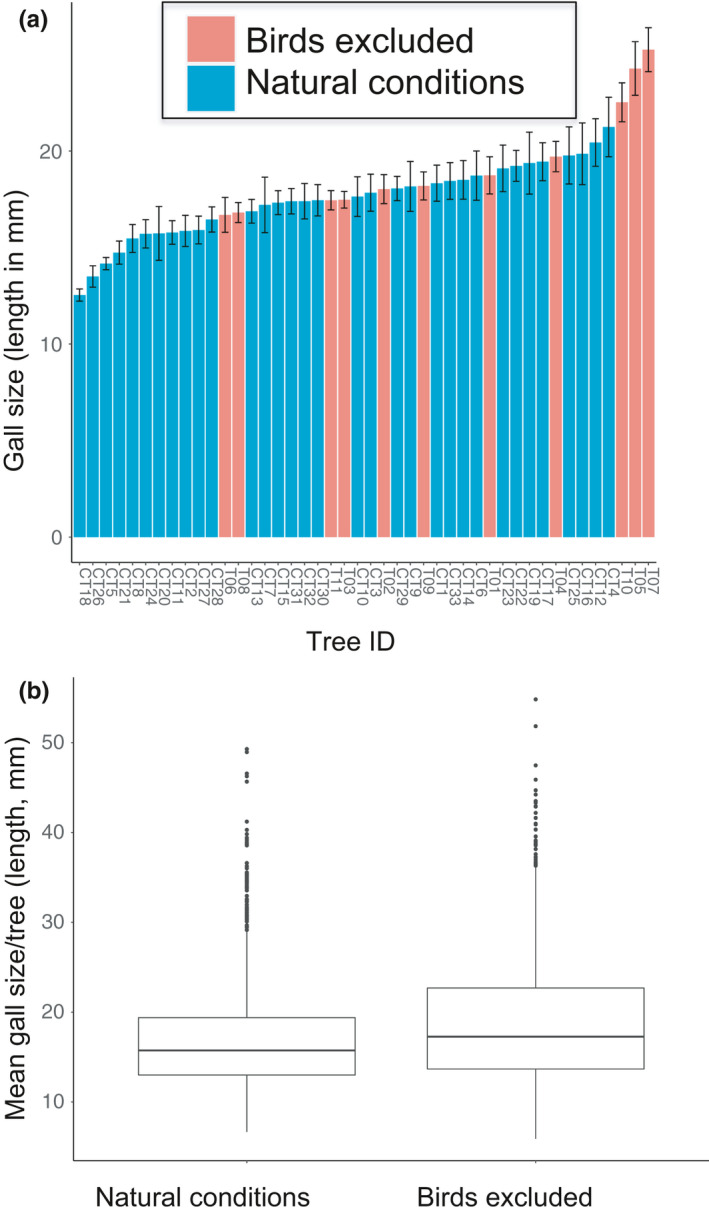
Mean gall size (±*SE*) arranged in rank order by length per tree for (a) 33 *Q. virginiana* under natural conditions (blue) and 11 *Q. virginiana* trees where birds had been excluded (orange). (b) Box plot comparison of mean gall size per tree under natural conditions and those experimentally protected from bird predation

In the absence of bird predation, we predicted that larger galls would evolve based on the association between gall size and chamber number where selection favors larger galls (Figure 2). Mean gall length (±*SE*) on trees where birds have been excluded for five years was 19.56 ± 0.23 mm, which was 12.3% larger than galls on trees exposed to bird predation under natural conditions (mean ± *SE* = 17.42 ± 0.19 mm) (Figure 4; Table 2). This difference was highly significant between bird predation treatments (*F*_df=1,43_ = 52.4, *p* < .0001; Figure 4b), even when controlling for the significant and strong differences between individual trees in gall size in the nested ANOVA (*F*_df=43,1,396_ = 7.2, *p* < .0001; Table 2). Notably, the three trees with the largest average gall size in this study (out of the 44 trees measured) were from the bird exclusion treatment, and seven of the eleven trees from the bird exclusion experiment exhibited larger galls than the average found under natural conditions (Figure 4a).

Given the observed change in phenotype over the five‐year study period of bird exclusion, we quantified these changes in terms of darwins and haldanes, which measure evolutionary change per unit time (or generation). On average, we observed a rate of evolutionary change in our experiment of 23.17 kilodarwins and 0.4252 haldanes, which are both high relative to other previously reported values of contemporary phenotypic change (Hendry & Kinnison, 1999).

## 4 DISCUSSION

Trade‐offs play an important role in understanding the evolution of phenotypes by considering the costs and benefits of adaptations in complex environments (Agrawal et al., 2010; Roff & Fairbairn, 2007). In this study, we document a fitness trade‐off between fecundity and survivorship for the gall‐forming wasp *Callirhytis quercusbatatoides* on its host plant *Quercus virginiana* associated with the trait gall size. Under natural conditions, there is a positive association between the trait gall size and offspring number, as larger galls contain more chambers and thus more individual wasps per gall (Figure 2). In contrast, there is also a negative association between gall size and survival, as larger galls are attacked by bird predators at a significantly higher rate than smaller galls (Figure 3). When bird predation was experimentally removed in our long‐term (five years) exclusion study, we observed a shift in gall size in the direction predicted from our observational experiments (Figure 4). Overall, our study demonstrates how two opposing forces of selection can generate a trade‐off acting on gall size, which is a critical trait for cynipid gall wasps (Stone & Cook, 1998; Stone & Schönrogge, 2003; Stone et al., 2002). Moreover, this study provides an example of rapid evolutionary change in response to human‐mediated habitat change on an ecological time‐scale (Carroll & Boyd, 1992; Carroll, Klassen, & Dingle, 1998).

### 4.1 Trade‐offs in phenotypes and stabilizing selection

Trade‐offs can take many forms: intrinsic resource trade‐offs where genotypes that produce more offspring can compromise investment in each individual offspring (Parker & Begon, 1986), or extrinsic trade‐offs, for example, when a trait provides a benefit in some environments, but can be maladaptive in others traits (Langerhans, Layman, & DeWitt, 2005). Our study has documented an example of an extrinsic, environment‐dependent trade‐off for a trait in gall wasps. Collectively, the opposing forces of selection, favoring larger galls to increase fecundity and favoring smaller galls to avoid predation by birds, generates an optimal gall size that is intermediate in the range of phenotypes resulting in a landscape‐wide pattern of stabilizing selection. Our study is similar to another well‐studied system, the *Eurosta* fly that induces galls on its host plant *Solidago* (Abrahamson, Sattler, McCrea, & Weis, 1989; Abrahamson & Weis, 1997; Weis, Abrahamson, & Andersen, 1992; Weis & Kapelinski, 1994). In this system, variation in gall size is associated with a fitness trade‐off between larger galls avoiding attack from parasitic insects (i.e., parasitoids), but being more likely to be attacked by birds, and smaller galls avoiding attack from birds, but being more likely to be attacked by parasitoid wasps. Our study does differ from studies of *Eurosta* in that we did not estimate selection resulting from parasitism, which can be a substantial source of mortality in some cynipid gall wasp species (Forbes et al., 2016). If patterns were similar among systems, then we would predict that larger galls would be adaptive for two reasons. First, as highlighted here, larger galls generate more offspring (Figure 2). Second, if similar to *Eurosta*, larger galls may reduce attack of parasitoid wasps. One way to test this in the future would be to compare parasitism rates between larger and smaller galls within trees, or between trees with larger and smaller sized galls on average.

### 4.2 Experimental studies of natural selection in the wild

Kingsolver et al. (2001) reviewed the strength of linear (and quadratic) phenotypic selection from the wild. In this review of 63 different studies, the authors found that directional selection was typically weak on average, but exhibited an exponential distribution with many strong estimates of directional selection observed in the tail of the distribution. Moreover, the review found that selection based on fecundity tended to be stronger that selection based on survival. Similarly, in our study, we found that directional selection for larger galls, based on fecundity, was strong, with our observed selection gradient (=0.74) over 4× greater than the average of all directional selection gradients reported in Kingsolver et al. (2001). Moreover, our estimate of selection due to fecundity was much greater than that based on survival in accord with the observations of average differences being higher for fecundity than survival in Kingsolver et al. (2001).

Hendry and Kinnison (1999) reviewed selected microevolution studies that calculated the rates of evolution change (i.e., the change in phenotypes over time). The authors concluded that many studies that measured experimentally induced microevolutionary change that span just a few generations observed “rapid” change. Our study similarly documented a strong shift in gall size over the “short” five‐year study period, which places our observed changes on the high end in the distribution of values estimating rates of evolutionary change (similar to those observed in Darwin's finches or Trinidadian guppies; Hendry & Kinnison, 1999). Collectively, this implies that response to environmental changes can be quite fast, predictable, and, in many cases, easily quantifiable when the underlying associations with phenotype and fitness are known (Grant & Grant, 1995).

### 4.3 Gall formers and studies of phenotypic selection in the wild

Galls are an extended manifestation of the wasp's phenotype (Dawkins, 1982), and among galling insects, there are many hypotheses regarding the adaptive function of the galls (Stone & Schönrogge, 2003; Stone et al., 2002). This morphological variation among cynipid galls includes sticky or hairy surfaces, size and color variation, spiked or thick walls, and even false chambers (Egan, Hood, Martinson, & Ott, 2018; Stone & Schönrogge, 2003). The leading hypothesis for the variation in gall morphology is protection against natural enemies (Stone & Schönrogge, 2003; Stone et al., 2002). Most gall wasp populations are attacked by large communities of natural enemies, including parasitoids, inquilines, and larger vertebrate predators, which as a whole can inflict high levels of mortality (sometimes as high as 99%; Egan et al., unpublished data). Our study is one of only a few to document vertebrate predation on galls including birds (Hails & Crawley, 1992; László et al., 2014; Schönrogge et al., 2013; Tscharntke, 1992) and squirrels (Shealer, Snyder, Dreisbach, Sunderlin, & Novak, 1999), which has rarely been studied in gall wasps (Stone et al., 2002), and further document the important role that vertebrates can plan in the evolution of gall morphology (Abrahamson & Weis, 1997). In addition to protection from natural enemies, gall formers receive nutrition from their host plant and protection from challenging environmental conditions. Each of these factors has likely played a role in the origin of gall formation, as well as the evolution of the great diversity of gall morphologies (Stone & Schönrogge, 2003).

We also find that individual trees vary in the mean gall sized produced and that selection from bird predation also varies by tree (Tables 1 and 2). The important role of variation in individual host plants in the interaction with gall‐forming insects has been documented in the live oak system associated with another gall‐forming wasp, *Belonocnema treatae*, where subpopulations have been shown to form demes locally adapted to individual plants (Egan & Ott, 2007) and individual trees exhibit different forms of selection on the leaf gall size induced by *B. treatae* (Egan et al., 2011). Moreover, the role of host plant genotype has been shown to be important in other gall former systems, such as the *Eurosta*‐*Solidago* system (Abrahamson & Weis, 1997). Thus, individual plants potentially represent different selective environments, which is consistent with the documented individual variation in defensive chemistry (Osier, Hwang, & Lindroth, 2000), phenology (Mopper, 2005), and biotic and abiotic setting (Henriksson et al., 2003).

### 4.4 Loss of species interactions changes evolutionary trajectory

Since the vast majority of the Earth's land surface (~80%) has been modified in some way for human use (Sanderson et al., 2002), the number of species interactions in terrestrial environments is rapidly decreasing and the multitrophic impacts of this loss are just beginning to be addressed (Hood et al., 2019; Johnson & Munshi‐South, 2017; Start et al., 2018). Removing or reducing bird predation is one example of these ecological disruptions. Rogers et al. (2012) find that bird loss on the island of Guam has had a cascading effect on spider populations, where webs were found to be 40× more common during the rainy season than comparable islands with birds (Rogers et al., 2012). Similar to Rogers et al. (2012), Hood et al. (2019) found that bird removal from live oaks in Houston, Texas, led to outbreak levels of one of the most venomous herbivorous caterpillars in North America. As woodpeckers have also been found to show marked decreases in abundance associated with human‐mediated habitat change (Conner & Rudolph, 1991), our experimental manipulation directly addresses the effects of the loss of birds in our region as well.

### 4.5 Caveats

Two related issues not considered in the current experimental design are the role of phenotypic plasticity and the genetic basis of gall size (Formiga, Silveira, Fernandes, & Isaias, 2015; László & Tóthmérész, 2013; Weis & Abrahamson, 1986; Weis et al., 1992). It is possible that plasticity plays a role in the process of gall formation, which is an interaction between the insect genome, the plant genome, and the environment (Weis & Abrahamson, 1986; Weis et al., 1992). While our comparison of netted and non‐netted trees is consistent with evolution by natural selection, it is possible that there were some undetectable environmental differences between trees that could account for unexplained variation in gall size. Trees at both sites (netted and non‐netted) are growing in similar semiurban conditions with trees at both sites growing in the proximity to buildings, sidewalks, roads, and other man‐made structures, suggesting that the location of trees is unlikely to influences our results. However, to further exclude this competing hypothesis in future work, we would need to perform a common garden experiment showing that wasps collected from bird exclusion trees do in fact induce larger galls than wasps from natural trees when exposed to a common plant genotype. We currently cannot do this experiment, because the alternative generation of this cynipid wasp remains unknown (see *Natural History* section in Section 2). In general, we believe our results are most consistent and parsimonious with rapid evolutionary change, but we cannot rule out additional contributions from unmeasured sources.

## 5 SUMMARY AND CONCLUSION

We document a fitness trade‐off between fecundity and survival in the gall‐forming wasp *C. quercusbatatoides* on its host plant *Quercus virginiana* where larger galls generate more offspring, but are also more likely to be attacked by birds. Conversely, smaller galls generate fewer offspring, but avoid predation by birds. We then compliment our observation study with an experiment where bird predators are excluded for five years and find that gall size has evolved to be 12% larger on average than control tree. Moreover, if you consider tiny insects that are short‐lived and are poor dispersers on very large, long‐lived host plants as individual populations, our results suggest that similar selective pressures have resulted in similar or parallel patterns of phenotypic response across a subset of the eleven experimental populations. Since all host‐associated populations of *C. quercusbatatoides* share a common regional gene pool, it is possible that most populations share similar standing genetic variation and architecture such that populations may respond similarly.

## CONFLICTS OF INTEREST

All authors confirm that they have no conflicts of interest.

## AUTHOR CONTRIBUTION

**Amanda Weaver:** Conceptualization (equal); Formal analysis (equal); Investigation (equal); Methodology (equal); Visualization (lead); Writing‐original draft (lead); Writing‐review & editing (equal). **Glen Hood:** Conceptualization (equal); Formal analysis (supporting); Investigation (equal); Methodology (equal); Supervision (equal); Validation (supporting); Visualization (supporting); Writing‐original draft (supporting); Writing‐review & editing (equal). **Michael P. Foster:** Conceptualization (supporting); Investigation (supporting); Methodology (supporting); Visualization (supporting); Writing‐review & editing (supporting). **Scott Egan:** Conceptualization (equal); Data curation (equal); Formal analysis (equal); Funding acquisition (equal); Investigation (equal); Methodology (equal); Project administration (lead); Resources (equal); Supervision (equal); Validation (equal); Visualization (equal); Writing‐original draft (lead); Writing‐review & editing (lead). 

## Supporting information

Supplementary Material

## Data Availability

Raw data from this study from our observational study and experiment are archived as.csv files with a Read_Me file at the Dryad Digital Repository (https://datadryad.org) located at https://doi.org/10.5061/dryad.d2547d81b.

## References

[ece36682-bib-0001] Abrahamson, W. G. , Sattler, J. F. , McCrea, K. D. , & Weis, A. E. (1989). Variation in selection pressures on the goldenrod gall fly and the competitive interactions of its natural enemies. Oecologia, 79, 15–22. 10.1007/BF00378234 28312807

[ece36682-bib-0002] Abrahamson, W. G. , & Weis, A. E. (1997). Evolutionary ecology across three trophic levels goldenrods, gallmakers, and natural enemies. Princeton, NJ: Princeton University Press.

[ece36682-bib-0003] Agrawal, A. A. , Conner, J. K. , & Rasmann, S. (2010). Tradeoffs and negative correlations in evolutionary ecology In: Bell M. A., Futuyma D. J., Eanes W. F., & Levinton J. S. (Eds.), Evolution since Darwin: The First 150 Years (pp. 243–268). Sunderland, Massachusetts: Sinauer Associates.

[ece36682-bib-0004] Ashmead, W. H. (1881). On the cynipidous galls of Florida. Transactions of the American Entomological Society, 9, xxiv–xxviii.

[ece36682-bib-0005] Berrigan, D. (1991). The allometry of egg size and number in insects. Oikos, 60, 313–321. 10.2307/3545073

[ece36682-bib-0006] Brodie III, E. D. , & Janzen, F. J. (1996). On the assignment of fitness values in statistical analyses of selection. Evolution, 50, 437–442. 10.1111/j.1558-5646.1996.tb04505.x 28568868

[ece36682-bib-0007] Brodie, E. D. III , Moore, J. A. , & Janzen, F. J. (1995). Visualizing and quantifying natural selection. Trends in Ecology and Evolution, 10, 313–318. 10.1016/S0169-5347(00)89117-X 21237054

[ece36682-bib-0008] Carroll, S. P. , & Boyd, C. (1992). Host race radiation in the soapberry bug: Natural history with the history. Evolution, 46, 1052–1069. 28564420 10.1111/j.1558-5646.1992.tb00619.x

[ece36682-bib-0009] Carroll, S. P. , Klassen, S. P. , & Dingle, H. (1998). Rapidly evolving adaptations to host ecology and nutrition in the soapberry bug. Evolution and Ecology, 12, 955–968.

[ece36682-bib-0010] Conner, R. N. , & Rudolph, D. C. (1991). Forest habitat loss, fragmentation, and red‐cockaded woodpecker populations. Wilson Bulletin., 103, 446–457.

[ece36682-bib-0011] Cook, L. , & Saccheri, I. (2013). The peppered moth and industrial melanism: Evolution of a natural selection case study. Heredity, 110(3), 207–212. 10.1038/hdy.2012.92 23211788 PMC3668657

[ece36682-bib-0012] Craig, T. P. , Itami, J. K. , & Horner, J. D. (2007). Geographic variation in the evolution and coevolution of a tritrophic interaction. Evolution, 61, 1137–1152. 10.1111/j.1558-5646.2007.00099.x 17492967

[ece36682-bib-0013] Dawkins, R. (1982). The Extended Phenotype. Oxford: Oxford University Press.

[ece36682-bib-0014] Dunn, J. C. , Halenar, L. B. , Davies, T. G. , Cristobal‐Azkarate, J. , Reby, D. , Sykes, D. , … Knapp, L. A. (2015). Evolutionary trade‐off between vocal tract and testes dimensions in howler monkeys. Current Biology, 25, 2839–2844. 10.1016/j.cub.2015.09.029 26592343 PMC4635310

[ece36682-bib-0015] Egan, S. P. , Hood, G. R. , Martinson, E. , & Ott, J. R. (2018). Quick Guide: Cynipid gall wasps. Current Biology, 28, PR1370–R1374 10.1016/j.cub.2018.10.028 30562523

[ece36682-bib-0016] Egan, S. P. , Hood, G. R. , & Ott, J. R. (2011). Natural selection on gall size: Variable contributions of individual host plant to population‐wide patterns. Evolution, 65, 3543–3557. 22133224 10.1111/j.1558-5646.2011.01396.x

[ece36682-bib-0017] Egan, S. P. , & Ott, J. R. (2007). Host plant quality and local adaptation determine the distribution of a gall forming herbivore. Ecology, 88, 2868–2879. 10.1890/06-1303.1 18051656

[ece36682-bib-0018] Endler, J. A. (1986). Natural selection in the wild. Princeton, NJ: Princeton University Press.

[ece36682-bib-0019] Engelhardt, G. P. (1946). The North American clearwing moths of the family Aegeriidae. U.S. National Museum Bulletin, 190, 1–222. 10.5479/si.03629236.190.1

[ece36682-bib-0020] Fischer, J. , & Lindenmayer, D. B. (2007). Landscape modification and habitat fragmentation: A synthesis. Global Ecology and Biogeography, 16, 265–280. 10.1111/j.1466-8238.2007.00287.x

[ece36682-bib-0021] Forbes, A. A. , Hood, G. R. , Hall, M. C. , Lund, J. , Izen, R. , Egan, S. P. , & Ott, J. R. (2016). Parasitoids, hyperparasitoids, and inquilines associated with the sexual and asexual generations of the gall former, Belonocnema treatae (Hymenoptera: Cynipidae). Annals of the Entomological Society of America, 109, 49–63.

[ece36682-bib-0022] Formiga, A. T. , Silveira, F. A. O. , Fernandes, G. W. , & Isaias, R. M. S. (2015). Phenotypic plasticity and similarity among gall morphotypes on a superhost, *Baccharis reticularia* (Asteraceae). Plant Biology, 17, 512–521. 25124804 10.1111/plb.12232

[ece36682-bib-0023] Gingerich, P. D. (1993). Quantification and comparison of evolutionary rates In Dodson P., & Gingerich P. D. (Eds.), Functional morphology and evolution, American Journal of Science (293A, pp. 453–478).

[ece36682-bib-0024] Grant, P. R. , & Grant, B. R. (1995). Predicting microevolutionary response to directional selection on heritable evolution. Evolution, 62, 845–856. 10.1111/j.1558-5646.1995.tb02236.x 28565006

[ece36682-bib-0025] Hails, R. , & Crawley, M. (1992). Spatial density dependence in populations of a cynipid gall‐former *Andricus quercuscalicis* . Journal of Animal Ecology, 61, 567–583.

[ece36682-bib-0026] Haldane, J. B. S. (1949). Suggestions as to quantitative measurement of rates of evolution. Evolution, 3, 51–56. 10.1111/j.1558-5646.1949.tb00004.x 18115117

[ece36682-bib-0027] Heath, J. J. , Abbot, P. , & Stireman, J. O. III (2018). Adaptive divergence in a defense symbiosis driven from the top down. American Naturalist, 192, E21–E36. 10.1086/697446 29897808

[ece36682-bib-0028] Hendry, A. P. (2017). Eco‐evolutionary dynamics. Princeton, NJ: Princeton University Press.

[ece36682-bib-0029] Hendry, A. P. , & Kinnison, M. T. (1999). The pace of modern life: Measuring rates of microevolution. Evolution, 53, 1637–1653. 28565449 10.1111/j.1558-5646.1999.tb04550.x

[ece36682-bib-0030] Henriksson, J. , Haukioja, E. , Ossipov, V. , Ossipova, S. , Sillanpää, S. , Kapari, S. L. , & Pihlaja, K. (2003). Effects of host shading on consumption and growth of the geometrid *Epirrita autumnata*: Interactive roles of water, primary and secondary compounds. Oikos, 103, 3–16.

[ece36682-bib-0031] Hood, G. R. , Comerford, M. , Weaver, A. K. , Morton, P. M. , & Egan, S. P. (2019). Human‐mediated disturbance in multitrophic interactions results in outbreak levels of North America’s most venomous caterpillar. Biology Letters, 15, 20190470 10.1098/rsbl.2019.0470 31480937 PMC6769147

[ece36682-bib-0032] Hood, G. R. , & Ott, J. R. (2010). Developmental plasticity and reduced susceptibility to natural enemies following host plant defoliation in a specialized herbivore. Oecologia, 162, 673–683. 10.1007/s00442-009-1492-9 19916027

[ece36682-bib-0033] Hood, G. R. , & Ott, J. R. (2017). Independent life history evolution between generations of bivoltine species: A case study of cyclical parthenogenesis. Oecologia, 183, 1053–1064. 10.1007/s00442-017-3824-5 28144732

[ece36682-bib-0034] Hood, G. R. , Zhang, L. , & Egan, S. P. (2018). Digest: Disentangled bank: Less diverse urban environments modify the shape and magnitude of natural selection. Evolution, 72, 1972–1973. 10.1111/evo.13577 30101493

[ece36682-bib-0035] Ito, M. , & Hijii, N. (2004). Roles of gall morphology in determining potential fecundity and avoidance of parasitoid attack in *Aphelonyx glanduliferae* . Journal of Forest Research, 9, 93–100.

[ece36682-bib-0036] Janzen, F. J. , & Stern, H. S. (1998). Logistic regression for empirical studies of multivariate selection. Evolution, 52, 1564–1571. 10.1111/j.1558-5646.1998.tb02237.x 28565316

[ece36682-bib-0037] Johnson, M. T. J. , & Munshi‐South, J. (2017). Evolution of life in urban environments. Science, 358, 1–11. 10.1126/science.aam8327 29097520

[ece36682-bib-0038] Kingsolver, J. G. , Hoekstra, H. E. , Hoekstra, J. M. , Berrigan, D. , Vignieri, S. N. , Hill, C. E. , … Beerli, P. (2001). The strength of phenotypic selection in natural populations. American Naturalist, 157, 245–261. 10.1086/319193 18707288

[ece36682-bib-0039] Lande, R. (1979). Quantitative genetic analysis of multivariate evolution, applied to brain:Body size allometry. Evolution, 37, 1210–1226. 10.1111/j.1558-5646.1979.tb04694.x 28568194

[ece36682-bib-0040] Lande, R. , & Arnold, S. J. (1983). The measurement of selection on correlated characters. Evolution, 37, 1210–1226. 10.1111/j.1558-5646.1983.tb00236.x 28556011

[ece36682-bib-0041] Langerhans, R. B. , Layman, C. A. , & DeWitt, T. J. (2005). Male genital size reflects a tradeoff between attracting mates and avoiding predators in two live‐bearing fish species. Proceedings of the National Academy of Sciences, 102, 7618–7623. 10.1073/pnas.0500935102 PMC1140428 15894618

[ece36682-bib-0042] László, Z. , Sólyom, K. , Prázsmári, H. , Barta, Z. , & Tóthmérész, B. (2014). Predation on rose galls: Parasitoids and predators determine gall size through directional selection. PLoS One, 9, e99806 10.1371/journal.pone.0099806 24918448 PMC4053394

[ece36682-bib-0043] László, Z. , & Tóthmérész, B. (2013). The enemy hypothesis: Correlates of gall morphology with parasitoid attack rates in two closely related rose cynipid galls. Bulletin of Entomological Research, 103, 326–335. 10.1017/S0007485312000764 23217451

[ece36682-bib-0044] Mitchell‐Olds, T. , & Shaw, R. G. (1987). Regression analysis of natural selection: Statistical inference and biological interpretation. Evolution, 41, 1149–1161. 10.1111/j.1558-5646.1987.tb02457.x 28563617

[ece36682-bib-0045] Mopper, S. (2005). Phenology—how time creates spatial structure in endophagous insect populations. Annales Zoologici Fennici, 42, 327–333.

[ece36682-bib-0046] Noyes, J. S. (2019). Universal Chalcidoidea Database. World Wide Web Electronic Publication. Retrieved from http://www.nhm.ac.uk/chalcidoids

[ece36682-bib-0047] Ohm, J. R. , & Miller, T. E. X. (2014). Balancing anti‐herbivore benefits and anti‐pollinator costs of defensive mutualists. Ecology, 95, 2924–2935. 10.1890/13-2309.1

[ece36682-bib-0048] Osier, T. L. , Hwang, S. Y. , & Lindroth, R. L. (2000). Within‐ and between‐year variation in early season phytochemistry of quaking aspen (*Populus tremuloides* Michx.) clones. Biochemical Systematics and Ecology, 28, 197–208. 10.1016/S0305-1978(99)00056-3

[ece36682-bib-0049] Parker, G. A. , & Begon, M. (1986). Optimal egg size and clutch size: Effects on environment and maternal phenotype. American Naturalist, 128, 573–592.

[ece36682-bib-0050] Parmesan, C. (2006). Ecological and evolutionary responses to recent climate change. Annual Review of Ecology, Evolution, and Systematics, 37, 637–669.

[ece36682-bib-0051] Price, P. , Abrahamson, W. G. , Hunter, M. , & Melika, G. (2004). Using gall wasps on oaks to test broad ecological concepts. Conservation Biology, 18, 1405–1416. 10.1111/j.1523-1739.2004.00547.x

[ece36682-bib-0052] Raupp, M. J. , Shrewsbury, P. M. , & Herms, D. A. (2010). Ecology of herbivorous arthropods in urban landscapes. Annual Review of Ecology, Evolution, and Systematics, 55, 19–38. 10.1146/annurev-ento-112408-085351 19961321

[ece36682-bib-0053] Reznick, D. N. , & Ghalambor, C. K. (2001). The population ecology of contemporary adaptations: What empirical studies reveal about the conditions that promote adaptive evolution. Genetica, 112, 183–198. 11838765

[ece36682-bib-0054] Roff, D. A. , & Fairbairn, D. J. (2007). The evolution of tradeoffs: Where are we? Journal of Evolutionary Biology, 20, 433–447. 10.1111/j.1420-9101.2006.01255.x 17305809

[ece36682-bib-0055] Rogers, H. , Hille Ris Lambers, J. , Miller, R. , & Tewksbury, J. J. (2012). ‘Natural experiment’ demonstrates top‐down control of spiders by birds on a landscape level. PLoS One, 7, e43446 10.1371/journal.pone.0043446 22970126 PMC3436874

[ece36682-bib-0056] Sanderson, E. W. , Malanding, J. , Levy, M. A. , Redford, K. H. , Wannebo, A. V. , & Woolmer, G. (2002). The human footprint and the last of the wild. BioScience, 52, 891–904. 10.1641/0006-3568(2002)052[0891:THFATL]2.0.CO;2

[ece36682-bib-0057] Schluter, D. (1988). Estimating the form of natural selection on a quantitative trait. Evolution, 42, 849–861. 10.1111/j.1558-5646.1988.tb02507.x 28581168

[ece36682-bib-0058] Schönrogge, K. , Begg, T. , & Stone, G. N. (2013). Native birds and alien insects: Spatial density dependence in songbird predation of invading oak gallwasps. PLoS One, 8, e53959 10.1371/journal.pone.0053959 23342048 PMC3544717

[ece36682-bib-0059] Seibold, S. , Cadotte, M. W. , MacIvor, J. S. , Thorn, S. , & Müller, J. (2018). The necessity of multitrophic approaches in community ecology. Trends in Ecology and Evolution, 33, 754–764. 10.1016/j.tree.2018.07.001 30146326

[ece36682-bib-0060] Shealer, D. A. , Snyder, J. P. , Dreisbach, V. C. , Sunderlin, D. F. , & Novak, J. A. (1999). Foraging patterns of eastern gray squirrels (*Sciurus carolinensis*) on goldenrod gall insects, a potentially important winter food resource. The American Midland Naturalist, 142, 102–109.

[ece36682-bib-0061] Start, D. , Bonner, C. , Weis, A. E. , & Gilbert, B. (2018). Consumer‐resource interactions along urbanization gradients drive natural selection. Evolution, 72, 1863–1873. 10.1111/evo.13544 29972241

[ece36682-bib-0062] Start, D. , Weis, A. E. , & Gilbert, B. (2019). Indirect interactions shape selection in a multispecies food web. American Naturalist, 193, 321–330. 10.1086/701785 30794449

[ece36682-bib-0063] Stone, G. N. , & Cook, J. M. (1998). The structure of cynipid oak galls: Patterns in the evolution of an extended phenotype. Proceedings of the Royal Society of London B: Biological Sciences, 265, 979–988. 10.1098/rspb.1998.0387

[ece36682-bib-0064] Stone, G. N. , & Schönrogge, K. (2003). The adaptive significance of insect gall morphology. Trends in Ecology and Evolution, 18, 512–522. 10.1016/S0169-5347(03)00247-7

[ece36682-bib-0065] Stone, G. N. , Schönrogge, K. , Atkinson, R. J. , Bellido, D. , & Pujade‐Villar, J. (2002). The population biology of oak gall wasps (Hymenoptera: Cynipidae). Annual Review of Entomology, 47, 633–668. 10.1146/annurev.ento.47.091201.145247 11729087

[ece36682-bib-0066] Tscharntke, T. (1992). Cascade effects among four trophic levels: Bird predation on galls affects density‐dependent parasitism. Ecology, 73, 1689–1698. 10.2307/1940020

[ece36682-bib-0067] Weis, A. E. , & Abrahamson, W. G. (1985). Potential selective pressures by parasitoids on the evolution of a plant–herbivore interaction. Ecology, 66, 1261–1269.

[ece36682-bib-0068] Weis, A. E. , & Abrahamson, W. G. (1986). Evolution of a host plant manipulation by gall makers: Ecological and genetic factors in the *Solidago‐Eurostra* interaction. American Naturalist, 127, 681–695.

[ece36682-bib-0069] Weis, A. , Abrahamson, W. , & Andersen, M. (1992). Variable selection on Eurosta's Gall Size, I: The extent and nature of variation in phenotypic selection. Evolution, 46, 1674–1697. 28567758 10.1111/j.1558-5646.1992.tb01161.x

[ece36682-bib-0070] Weis, A. E. , & Gorman, W. L. (1990). Measuring selection on reaction norms: An exploration of the *Eurostra‐Solidao* system. Evolution, 44, 820–831. 28569024 10.1111/j.1558-5646.1990.tb03807.x

[ece36682-bib-0071] Weis, A. , & Kapelinski, A. (1994). Variable selection on Eurosta's Gall Size. II. A path analysis of the ecological factors behind selection. Evolution, 48, 734–745. 28568264 10.1111/j.1558-5646.1994.tb01357.x

