# Supplementary material for: Trade‐off between fecundity and survival generates stabilizing selection on gall size
Source: Ecol Evol. 2020 Aug 17;10(18):10207–18. doi: 10.1002/ece3.6682 (PMC7520187; doi:10.1002/ece3.6682)
Supplement: Supplementary file 1 — Supplementary Material [file ECE3-10-10207-s001.docx]

Supplemental has been collapsed into the Methods section of the Main Text,

as per the request of the Associate Editor.
